# Effects of embedded micro-mindfulness on psychological symptoms in patients with advanced cancer receiving palliative care: a prospective controlled study

**DOI:** 10.3389/fonc.2026.1916107

**Published:** 2026-08-26

**Authors:** Qin Zhang, Qianer Li, Aiping Deng, Dan Yang, Dan Jia

**Affiliations:** 1 Outpatient Department, West China Hospital, Sichuan University, Chengdu, China; 2 Lung Cancer Center, West China Hospital, Sichuan University, Chengdu, China

**Keywords:** advanced cancer, anxiety and depression, micro-mindfulness, palliative care, psychological symptoms

## Abstract

**Objective:**

To evaluate changes in psychological distress, anxiety and depressive symptoms, sleep problems, and pain intensity associated with participation in an embedded micro-mindfulness program among patients with advanced cancer receiving palliative care, and to explore factors associated with psychological symptom response.

**Methods:**

This single-center, prospective, non-randomized observational study included 243 patients with advanced cancer receiving palliative care at West China Hospital. Patients who participated in the departmental micro-mindfulness program in addition to usual care comprised the MM group (n = 121), whereas those who received usual care without participating in the program comprised the UC group (n = 122). Outcomes were assessed at baseline and weekly for 4 weeks. Primary outcomes were anxiety and depressive symptoms assessed using the GAD-7 and PHQ-9, respectively. Secondary outcomes included psychological distress assessed using the Distress Thermometer (DT), sleep problems assessed using the two-item Insomnia Severity Index (ISI-2), and pain intensity assessed using the Numeric Rating Scale (NRS).

**Results:**

Baseline characteristics were comparable between groups. Significant group × time interactions were observed for GAD-7 (P = 0.001), PHQ-9 (P = 0.002), DT (P < 0.001), and ISI-2 (P < 0.001), with greater reductions over time observed in the MM group. The group × time interaction for NRS was not significant (P = 0.371). In the MM group, 73 patients (60.3%) achieved psychological symptom response.

**Conclusion:**

Embedded micro-mindfulness was associated with greater improvements in anxiety, depressive symptoms, psychological distress, and sleep problems, but not pain intensity. These findings support further evaluation of this brief program as a supportive-care strategy for patients with advanced cancer receiving palliative care.

## 1 Introduction

Patients with advanced cancer often experience substantial psychological and symptom burden during palliative care. In addition to physical symptoms such as pain, fatigue, and sleep disturbance, anxiety, depression, and psychological distress are also common and may further affect quality of life, treatment adherence, symptom perception, and caregiver burden (1–3). Therefore, psychological support is regarded as an important component of palliative care, with the goal of not only alleviating physical symptoms such as pain, fatigue, dyspnea, and sleep disturbance but also reducing psychological suffering, maintaining dignity, and improving patients’ overall experience during the advanced stage of illness (4–6).

Mindfulness-based interventions are psychological approaches that emphasize awareness of present-moment experiences, acceptance of emotions, and reduced reactive coping (7, 8). Previous studies have shown that mindfulness-based interventions may improve anxiety, depression, sleep problems, and overall psychological distress in patients with cancer (9–11). However, traditional mindfulness programs usually require a relatively long training period, fixed session schedules, and continuous guidance from trained professionals, such as multi-week group-based courses or structured programs (12, 13). For patients with advanced cancer receiving palliative care, reduced physical strength, limited hospitalization time, fluctuating symptom burden such as pain, fatigue, dyspnea, and sleep disturbance, and insufficient psychosocial care resources may limit the implementation and dissemination of traditional mindfulness interventions in routine clinical practice (14, 15).

Micro-mindfulness may provide a more feasible form of psychological support in palliative care settings. Compared with traditional mindfulness programs, micro-mindfulness emphasizes brief, low-burden, and repeatable practices that can usually be completed within a few minutes and embedded into routine care (16, 17). Through brief breathing awareness, body scan, or loving-kindness phrases, patients may receive immediate emotional regulation support within limited clinical contact time (18). For patients with advanced cancer, this approach requires less time and physical effort and may be more easily introduced by nurses or physicians at the bedside and continued after discharge with audio materials and online follow-up support (19, 20).

Although micro-mindfulness is theoretically suitable for palliative care settings, its feasibility and effects in patients with advanced cancer require further evaluation. Evidence remains limited regarding whether brief mindfulness-based practices embedded into routine palliative care can improve psychological distress, anxiety and depressive symptoms, sleep problems, and pain intensity in real-world clinical settings (21, 22). In addition, patients may respond differently to micro-mindfulness interventions, and identifying factors associated with psychological symptom improvement may help optimize patient selection and individualized psychosocial support (23).

Therefore, this study aimed to evaluate the effects of an embedded micro-mindfulness intervention in patients with advanced cancer receiving palliative care. Specifically, we examined changes in psychological distress, anxiety and depressive symptoms, sleep problems, and pain intensity, and further explored clinical and sociodemographic factors associated with psychological symptom response.

## 2 Methods

### 2.1 Study design and participants

This was a single-center, prospective, non-randomized observational study conducted among patients with advanced cancer receiving palliative care at West China Hospital between March 2025 and February 2026. During the study period, an embedded micro-mindfulness program was routinely available as an optional component of supportive care in the department. Participation in the program was voluntary. Patients who participated in the micro-mindfulness program in addition to usual care comprised the micro-mindfulness (MM) group, whereas those who did not participate in the program and continued to receive usual palliative care comprised the usual care (UC) group. The study evaluated changes in psychological distress, anxiety and depressive symptoms, sleep problems, and pain intensity, and explored clinical and sociodemographic factors associated with psychological symptom response.

Patients were eligible if they met the following criteria: (1) age ≥18 years; (2) diagnosed clinically or pathologically with an unresectable locally advanced, recurrent, or metastatic solid tumor for which curative treatment was no longer considered feasible; (3) receiving palliative care or care primarily focused on disease control, symptom management, and quality-of-life improvement; and (4) able to understand the study procedures and complete questionnaire assessments. Written informed consent was obtained from all participants before enrollment. Patients were excluded if they had severe cognitive impairment, impaired consciousness, or communication difficulties that prevented questionnaire completion; severe frailty or clinical deterioration that precluded participation; or missing key baseline data. Severe frailty was defined as an Eastern Cooperative Oncology Group (ECOG) performance status of 4. Clinical deterioration was defined as an acute or marked worsening of the patient’s clinical condition that, in the judgment of the treating physician, precluded completion of the baseline assessment or participation in the brief mindfulness practice. Cognitive and communication capacity was assessed during eligibility screening by a trained nurse or physician through a clinical interview. Patients were considered capable of completing the study instruments if they could understand the study information, communicate appropriately with the assessor, and complete the questionnaires.

**Figure 1:**
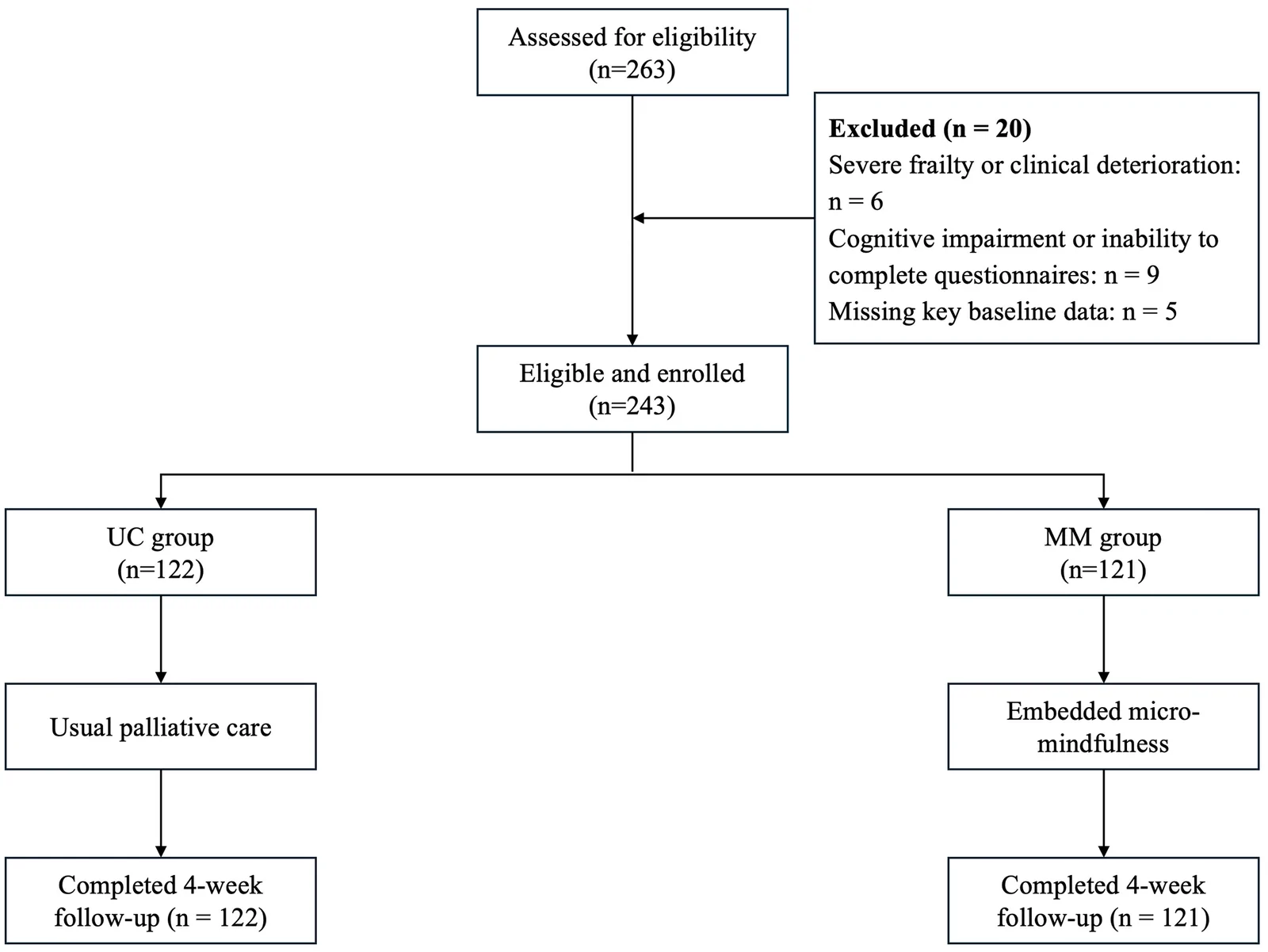
Flow diagram of participant enrollment and group formation.

A total of 263 patients were assessed for eligibility. Of these, 20 were excluded because of severe frailty or clinical deterioration, cognitive impairment or inability to complete questionnaires, refusal to participate or lack of informed consent, or missing key baseline data. Finally, 243 eligible patients were included in the analysis, including 122 patients in the UC group and 121 patients in the MM group. The participant flow is shown in **Figure 1**. The study was approved by the Ethics Committee of West China Hospital, Sichuan University [Approval No. 2025 Review (530)]. All participants provided informed consent, and the study was conducted in accordance with the Declaration of Helsinki.

### 2.2 Usual care and micro-mindfulness intervention

The UC group received usual palliative care, including symptom assessment and management, pain control, nutritional support, psychological support, nursing guidance, and necessary antitumor or supportive treatments. Specific treatment and nursing plans were adjusted by physicians and nurses according to each patient’s clinical condition, symptom burden, and individual needs.

Patients in the MM group participated in the departmental micro-mindfulness program in addition to usual palliative care. Each micro-mindfulness session consisted of three components performed sequentially and lasted approximately 5 minutes in total. First, a three-step breathing space lasting approximately 3 minutes guided patients to become aware of their current thoughts, emotions, and bodily sensations, focus attention on the natural rhythm and sensations of breathing, and then broaden their awareness to the body as a whole. This was followed by an approximately 1-minute brief body scan, during which patients sequentially directed attention to bodily sensations without judgment or attempting to change them. Finally, an approximately 1-minute loving-kindness practice was performed, during which patients silently repeated brief phrases intended to foster calmness, self-kindness, and goodwill toward themselves and others.

The program was delivered by nurses and physicians who had completed at least 4 hours of standardized training before program implementation. The training covered the rationale and procedures of the three micro-mindfulness components, bedside guidance procedures, use of standardized audio materials, and communication with patients during practice. During the initial bedside session, a trained nurse or physician introduced the complete practice sequence and guided the patient through all three components. During hospitalization, patients received at least two bedside guidance sessions to familiarize themselves with the practice sequence and facilitate subsequent self-practice. Standardized audio recordings corresponding to the three practice components were provided for self-practice. After discharge, patients received daily reminders and follow-up support through an online follow-up platform and were encouraged to complete the approximately 5-minute practice sequence once daily, with the timing adjusted flexibly according to their physical condition and symptom burden. The use of standardized training, guidance procedures, and audio materials was intended to ensure consistency in program delivery.

Practice adherence was monitored using daily completion records. During hospitalization, completion was documented by the nurse or physician providing bedside guidance, whereas after discharge, participants submitted photo-based check-ins through the online follow-up platform after completing each practice session (accessible only to authorized study personnel and used solely to document practice completion). Participants were scheduled to complete one session per day for 4 weeks (28 sessions in total). Adherence was calculated as the proportion of scheduled sessions with documented completion.

### 2.3 Outcome assessment

Outcome assessments were conducted at five time points: baseline (T0), week 1 (T1), week 2 (T2), week 3 (T3), and week 4 (T4). The primary outcomes were anxiety and depressive symptoms, assessed using the Generalized Anxiety Disorder-7 (GAD-7) and Patient Health Questionnaire-9 (PHQ-9), respectively. Higher GAD-7 and PHQ-9 scores indicated more severe anxiety and depressive symptoms. Secondary outcomes included psychological distress assessed using the Distress Thermometer (DT), sleep problems assessed using the two-item version of the Insomnia Severity Index (ISI-2), and pain intensity assessed using the Numeric Rating Scale (NRS). Psychological symptom response was defined as a reduction of at least 4 points in the GAD-7 or at least 5 points in the PHQ-9 from T0 to T4, based on previously reported minimal clinically important differences for these scales (24, 25). This definition was used to identify patients who achieved clinically meaningful improvement in anxiety or depressive symptoms.

### 2.4 Data collection

Baseline demographic, socioeconomic, and disease-related data were collected at enrollment. Demographic and socioeconomic variables included age, sex, body mass index (BMI), education level, marital status, medical payment type, and monthly household income. Disease-related variables included cancer category, ECOG performance status, and opioid analgesic use. ECOG performance status ranges from 0 (fully active) to 4 (completely disabled), with higher scores indicating greater functional impairment. Cancer categories were classified according to the primary tumor site as lung cancer, digestive system cancers, breast cancer, and other solid tumors. Lung, digestive system, and breast cancers were presented separately because they represented the major cancer categories in the study population, whereas less frequent tumor types were grouped as other solid tumors. Symptom-related assessments, including DT, GAD-7, PHQ-9, ISI-2, and NRS scores, were collected at baseline and at each follow-up time point according to the predefined assessment schedule.

### 2.5 Statistical analysis

Continuous variables were presented as means ± standard deviations or medians with interquartile ranges, depending on data distribution. Categorical variables were presented as numbers and percentages. Baseline characteristics were compared between the UC and MM groups using the independent-samples t-test or Mann–Whitney U test for continuous variables, and the chi-square test or Fisher’s exact test for categorical variables, as appropriate. Linear mixed-effects models were used to evaluate changes in GAD-7, PHQ-9, DT, ISI-2, and NRS scores from T0 to T4. Group, time, and the group × time interaction were included as fixed effects, and a random intercept for each participant was included to account for within-subject correlations across repeated measurements. The group × time interaction was used to determine whether changes in outcome scores over time differed between the UC and MM groups. In addition, between-group comparisons at each time point were performed to descriptively compare outcome scores between the two groups.

To explore factors associated with psychological symptom response after the micro-mindfulness intervention, logistic regression analysis was performed in the MM group. Psychological symptom response was defined as a reduction of at least 4 points in GAD-7 or at least 5 points in PHQ-9 from T0 to T4. Univariable logistic regression analysis was first performed, and variables with P < 0.10 in the univariable analysis, together with clinically relevant variables, were entered into the multivariable logistic regression model. Results were reported as odds ratios (ORs) with 95% confidence intervals (CIs). All statistical tests were two-sided, and P < 0.05 was considered statistically significant. Statistical analyses were performed using R version 4.5.1.

## 3 Results

### 3.1 Baseline characteristics

A total of 243 patients were included in the analysis, including 122 in the UC group and 121 in the MM group. All participants completed the scheduled assessments from T0 to T4. In the MM group, all 121 participants completed all 28 scheduled practice sessions, corresponding to an average frequency of 7 sessions per week and an adherence rate of 100%, based on daily completion records, including photo-based check-ins after discharge. Baseline disease and symptom burden were characterized by clinical characteristics and symptom assessment scores. ECOG performance status ≥2 was observed in 59.0% of the UC group and 54.5% of the MM group, while opioid analgesics were used by 55.7% and 51.2%, respectively. Median baseline scores in the UC and MM groups were 6.0 and 7.0 for DT, 9.0 and 9.0 for GAD-7, 11.0 and 10.0 for PHQ-9, 4.0 and 5.0 for ISI-2, and 5.0 and 5.0 for NRS, respectively. No significant between-group differences were observed in these baseline measures (all P > 0.05; **Table 1**).

**Table 1:** Baseline characteristics of participants.

Baseline characteristic	UC group (n = 122)	MM group (n = 121)	P
Age, years, mean (SD)	64.3 ± 10.6	65.5 ± 9.4	0.353
Sex, n (%)			0.566
Male	68 (55.7)	63 (52.1)	
Female	54 (44.3)	58 (47.9)	
BMI, kg/m², mean (SD)	22.7 ± 2.7	22.1 ± 3.2	0.111
Education level, n (%)			0.777
Junior high school or below	60 (49.2)	54 (44.6)	
Senior high school	37 (30.3)	40 (33.1)	
College or above	25 (20.5)	27 (22.3)	
Marital status, n (%)			0.528
Married/partnered	94 (77.0)	89 (73.6)	
Unmarried/divorced/widowed	28 (23.0)	32 (26.4)	
Medical payment type, n (%)			0.445
Medical insurance	92 (75.4)	86 (71.1)	
Self-payment	30 (24.6)	35 (28.9)	
Monthly household income, n (%)			0.759
<5,000 CNY	53 (43.4)	48 (39.7)	
5,000–9,999 CNY	45 (36.9)	45 (37.2)	
≥10,000 CNY	24 (19.7)	28 (23.1)	
Cancer category, n (%)			0.955
Lung cancer	37 (30.3)	33 (27.3)	
Digestive system cancers	57 (46.7)	58 (47.9)	
Breast cancer	20 (16.4)	22 (18.2)	
Other solid tumors	8 (6.6)	8 (6.6)	
ECOG performance status, n (%)			0.482
ECOG 0–1	50 (41.0)	55 (45.5)	
ECOG ≥2	72 (59.0)	66 (54.5)	
Opioid analgesic use, n (%)			0.482
No	54 (44.3)	59 (48.8)	
Yes	68 (55.7)	62 (51.2)	
Baseline DT, median (IQR)	6.0 (5.0-7.0)	7.0 (5.0-8.0)	0.345
Baseline GAD-7, median (IQR)	9.0 (7.2-11.0)	9.0 (7.0-12.0)	0.543
Baseline PHQ-9, median (IQR)	11.0 (8.2-14.0)	10.0 (8.0-13.0)	0.348
Baseline ISI-2, median (IQR)	4.0 (3.0-6.0)	5.0 (4.0-6.0)	0.294
Baseline NRS, median (IQR)	5.0 (4.0-6.0)	5.0 (4.0-6.0)	0.168

### 3.2 Changes in outcomes over time

Linear mixed-effects models showed significant time effects for all five outcomes across T0–T4. Significant group × time interactions were observed for GAD-7, PHQ-9, DT, and ISI-2, indicating greater reductions over time in the MM group, whereas the interaction for NRS was not statistically significant. Detailed estimates of changes and between-group differences are presented in **Table 2**.

**Table 2:** Linear mixed-effects model results and changes in outcomes over time.

Outcome	UC change	MM change	Between-group difference (95% CI)	Time effect P	Group effect P	Interaction P
GAD-7	−1.36	−2.71	−1.35 (−2.06 to −0.64)	<0.001	0.373	0.001
PHQ-9	−1.47	−2.69	−1.22 (−1.96 to −0.48)	<0.001	0.248	0.002
DT	−0.85	−2.08	−1.23 (−1.76 to −0.70)	<0.001	0.981	<0.001
ISI-2	−0.59	−1.52	−0.93 (−1.35 to −0.51)	<0.001	0.432	<0.001
NRS	−0.50	−0.63	−0.13 (−0.48 to 0.22)	0.007	0.101	0.371

Changes were calculated as T4 minus T0. Between-group differences were calculated as MM minus UC. Negative values indicate reductions in symptom scores, with more negative between-group differences indicating greater reductions in the MM group relative to the UC group. Interaction P represents the overall group × time interaction across T0–T4.

#### 3.2.1 Changes in anxiety and depressive symptoms

**Figure 2:**
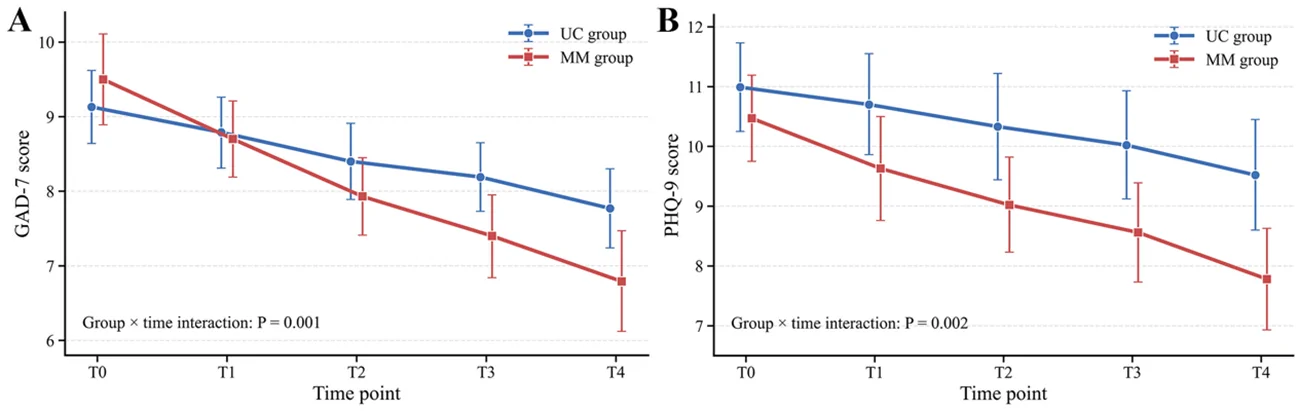
Changes in anxiety and depressive symptoms over time. **(A)** Changes in GAD-7 scores; **(B)** Changes in PHQ-9 scores.

Anxiety and depressive symptoms improved over time in both groups, with greater reductions observed in the MM group (**Table 3**). For GAD-7, the mean score decreased from 9.13 at T0 to 7.77 at T4 in the UC group, and from 9.50 to 6.79 in the MM group. Between-group comparisons showed no significant difference at baseline, whereas the MM group had significantly lower GAD-7 scores than the UC group at T3 and T4 (**Figure 2A**). The group × time interaction was significant (P = 0.001), indicating a greater reduction in anxiety symptoms in the MM group.

**Table 3:** Longitudinal changes in GAD-7 and PHQ-9 scores.

Group	T0	T1	T2	T3	T4
GAD-7					
UC	9.13 (8.64-9.62)	8.79 (8.31-9.26)	8.40 (7.89-8.91)	8.19 (7.73-8.65)	7.77 (7.24-8.30)
MM	9.50 (8.89-10.11)	8.70 (8.19-9.21)	7.93 (7.41-8.45)	7.40 (6.84-7.95)	6.79 (6.12-7.47)
P value	0.362	0.813	0.209	0.033	0.026
PHQ-9					
UC	10.99 (10.25-11.73)	10.70 (9.86-11.55)	10.33 (9.44-11.22)	10.02 (9.12-10.93)	9.52 (8.60-10.45)
MM	10.47 (9.75-11.19)	9.63 (8.76-10.50)	9.02 (8.23-9.82)	8.56 (7.73-9.39)	7.78 (6.93-8.63)
P value	0.324	0.082	0.033	0.021	0.007

A similar pattern was observed for depressive symptoms. The mean PHQ-9 score decreased from 10.99 at T0 to 9.52 at T4 in the UC group, and from 10.47 to 7.78 in the MM group. There was no significant between-group difference at baseline, while PHQ-9 scores were significantly lower in the MM group from T2 to T4 (**Figure 2B**). The group × time interaction was also significant (P = 0.002), suggesting greater improvement in depressive symptoms in the MM group.

#### 3.2.2 Changes in psychological distress

**Figure 3:**
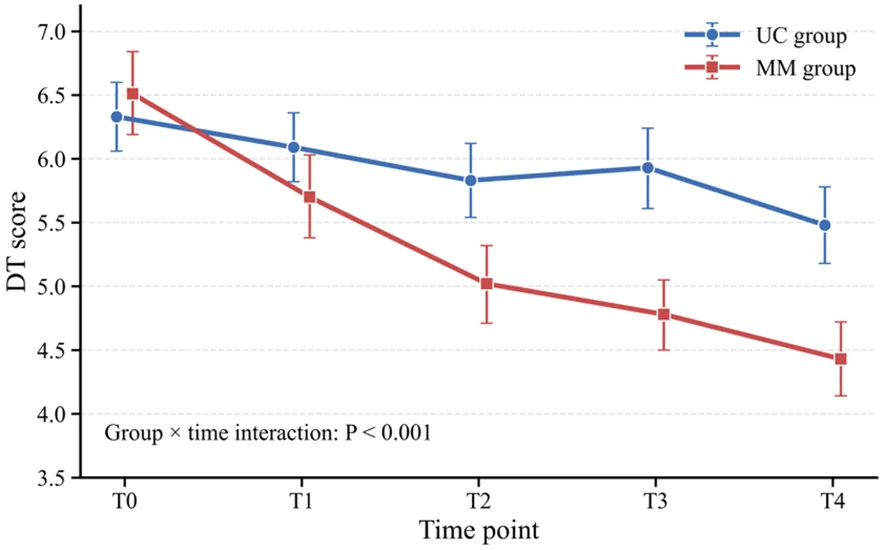
Changes in psychological distress assessed by DT over time.

Psychological distress decreased over time in both groups, with a more pronounced reduction in the MM group (**Table 4**; **Figure 3**). The mean DT score decreased from 6.33 at T0 to 5.48 at T4 in the UC group, corresponding to a reduction of 0.85 points. In the MM group, the mean DT score decreased from 6.51 to 4.43, corresponding to a reduction of 2.08 points. There was no significant between-group difference at baseline, whereas DT scores were significantly lower in the MM group from T2 to T4. The group × time interaction was significant (P < 0.001), indicating greater improvement in psychological distress in the MM group.

**Table 4:** Longitudinal changes in DT scores.

Group	T0	T1	T2	T3	T4
UC	6.33 (6.06-6.60)	6.09 (5.82-6.36)	5.83 (5.54-6.12)	5.93 (5.61-6.24)	5.48 (5.18-5.78)
MM	6.51 (6.19-6.84)	5.70 (5.38-6.03)	5.02 (4.71-5.32)	4.78 (4.50-5.05)	4.43 (4.14-4.72)
P value	0.393	0.074	<0.001	<0.001	<0.001

#### 3.2.3 Changes in sleep problems

**Figure 4:**
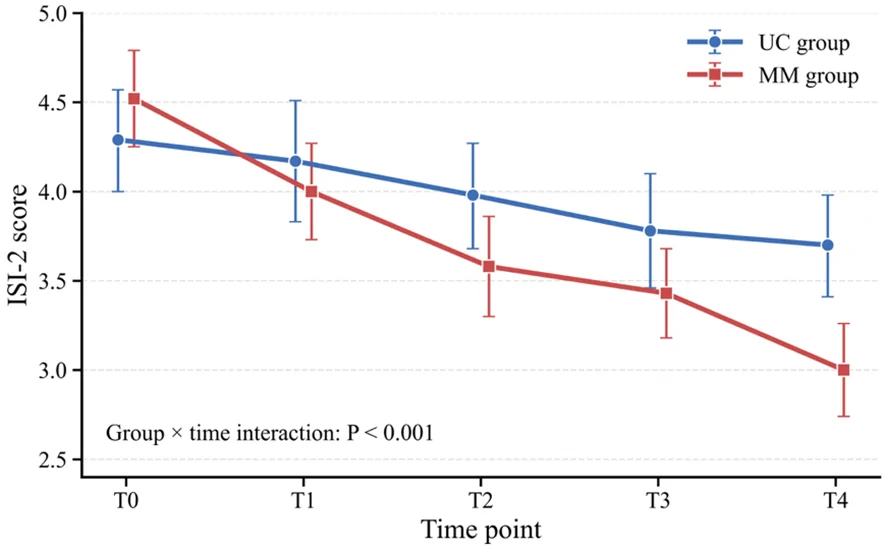
Changes in sleep problems assessed by ISI-2 over time.

Sleep problems decreased over time in both groups, with a greater reduction observed in the MM group (**Table 5**). The mean ISI-2 score decreased from 4.29 at T0 to 3.70 at T4 in the UC group, corresponding to a reduction of 0.59 points. In the MM group, the mean ISI-2 score decreased from 4.52 to 3.00, corresponding to a reduction of 1.52 points. There was no significant between-group difference from T0 to T3, whereas the MM group had significantly lower ISI-2 scores than the UC group at T4 (**Figure 4**). The group × time interaction was significant (P < 0.001), suggesting greater improvement in sleep problems in the MM group.

**Table 5:** Longitudinal changes in ISI-2 scores.

Group	T0	T1	T2	T3	T4
UC	4.29 (4.00-4.57)	4.17 (3.83-4.51)	3.98 (3.68-4.27)	3.78 (3.46-4.10)	3.70 (3.41-3.98)
MM	4.52 (4.25-4.79)	4.00 (3.73-4.27)	3.58 (3.30-3.86)	3.43 (3.18-3.68)	3.00 (2.74-3.26)
P value	0.25	0.437	0.059	0.091	<0.001

#### 3.2.4 Changes in pain intensity

**Figure 5:**
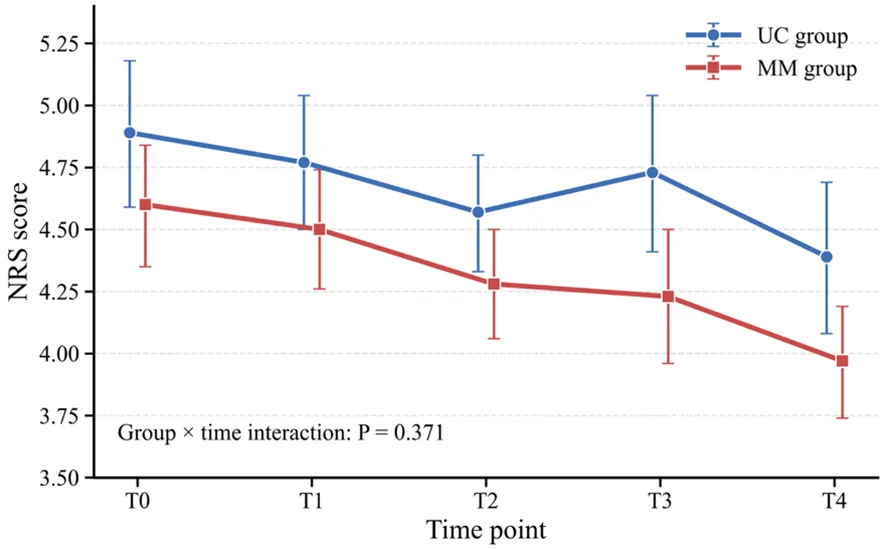
Changes in pain intensity assessed by NRS over time.

Pain intensity showed a modest decrease over time in both groups (**Table 6**; **Figure 5**). The mean NRS score decreased from 4.89 at T0 to 4.39 at T4 in the UC group, corresponding to a reduction of 0.50 points. In the MM group, the mean NRS score decreased from 4.60 to 3.97, corresponding to a reduction of 0.63 points. Between-group comparisons showed no significant differences from T0 to T2, while NRS scores were lower in the MM group at T3 and T4. However, although the overall time effect was significant (P = 0.007), the group × time interaction was not statistically significant (P = 0.371), indicating that the longitudinal change in pain intensity did not differ significantly between the two groups.

**Table 6:** Longitudinal changes in NRS scores.

Group	T0	T1	T2	T3	T4
UC	4.89 (4.59-5.18)	4.77 (4.50-5.04)	4.57 (4.33-4.80)	4.73 (4.41-5.04)	4.39 (4.08-4.69)
MM	4.60 (4.35-4.84)	4.50 (4.26-4.74)	4.28 (4.06-4.50)	4.23 (3.96-4.50)	3.97 (3.74-4.19)
P value	0.138	0.15	0.083	0.02	0.03

### 3.3 Factors associated with psychological symptom response

Among the 121 patients in the MM group, 73 patients (60.3%) achieved psychological symptom response, whereas 48 patients (39.7%) did not. Logistic regression analysis was performed to explore factors associated with psychological symptom response in the MM group (**Table 7**). In univariable analysis, age, monthly household income, cancer category, and ECOG performance status were associated with psychological symptom response. Higher monthly household income was associated with greater odds of response (OR = 2.98, 95% CI: 1.36–6.53, P = 0.006), whereas digestive system cancers (OR = 0.42, 95% CI: 0.20–0.88, P = 0.021) and ECOG ≥2 (OR = 0.32, 95% CI: 0.15–0.69, P = 0.004) were associated with lower odds of response. Older age was also associated with lower odds of response in univariable analysis (OR = 0.67, 95% CI: 0.45–0.99, P = 0.046). In multivariable analysis, monthly household income ≥5,000 CNY remained independently associated with higher odds of psychological symptom response (OR = 2.42, 95% CI: 1.04–5.63, P = 0.041). ECOG ≥2 remained independently associated with lower odds of response (OR = 0.39, 95% CI: 0.17–0.88, P = 0.023). Age, marital status, and cancer category were not independently associated with response after adjustment.

**Table 7:** Logistic regression analysis of factors associated with psychological symptom response.

Variable	Univariable OR (95% CI)	P	Multivariable OR (95% CI)	P
Age, year	0.67 (0.45–0.99)	0.046	0.76 (0.49–1.18)	0.224
Male sex	0.57 (0.27–1.20)	0.137		
BMI, kg/m²	1.03 (0.91–1.17)	0.643		
College or above	1.72 (0.67–4.45)	0.261		
Married/partnered	2.16 (0.96–4.87)	0.063	1.64 (0.67–4.02)	0.281
Self-payment	0.50 (0.23–1.09)	0.082		
Income ≥5,000 CNY	2.98 (1.36–6.53)	0.006	2.42 (1.04–5.63)	0.041
Digestive system cancers	0.42 (0.20–0.88)	0.021	0.57 (0.25–1.30)	0.181
ECOG ≥2	0.32 (0.15–0.69)	0.004	0.39 (0.17–0.88)	0.023
Opioid analgesic use	0.77 (0.37–1.61)	0.487		

## 4 Discussion

The overall pattern of findings suggests that embedded micro-mindfulness may be more relevant to psychological and sleep-related symptom management than to pain control in patients with advanced cancer receiving palliative care. Recent randomized studies of brief mindful breathing have shown that relatively short practices can produce rapid changes in selected psychological and physical symptoms in patients with cancer or advanced cancer (26, 27). Although these breathing-focused protocols differed from the present program in duration, intensity, and intervention content, they support the potential value of shorter mindfulness practices in populations with substantial symptom burden. The present program combined a three-step breathing space, brief body scan, and loving-kindness practice within an approximately 5-minute daily session repeated over 4 weeks. Differences in intervention content, intensity, and targeted symptoms may partly account for variation in outcomes across studies. In particular, the absence of a significant between-group difference in pain in the present study suggests that the potential benefits of brief mindfulness practices may not extend uniformly across symptom domains.

The observed improvements in GAD-7, PHQ-9, and DT scores suggest that embedded micro-mindfulness may help reduce emotional distress in patients with advanced cancer. This finding is broadly consistent with a randomized controlled trial in patients with advanced cancer, in which a 7-day mindful gratitude journaling intervention was associated with improvements in psychological distress and suffering (28). Although the intervention format differed from the present program, both approaches used brief, repeated practices intended to modify patients’ responses to distressing experiences. Psychological distress in palliative care may be shaped by symptom burden, uncertainty about disease progression, loss of autonomy, and existential concerns (29–31). Several complementary psychological processes may contribute to the observed associations. The three-step breathing space may redirect attention from distressing thoughts toward present-moment bodily and respiratory experience, thereby interrupting repetitive negative thinking such as worry and rumination. At the same time, observing thoughts and emotions without immediately reacting to or attempting to suppress them may promote acceptance and reduce emotional reactivity (32, 33). The loving-kindness component may provide an additional pathway by encouraging a less self-critical and more compassionate response to difficult experiences. Collectively, these processes may reduce the cognitive and emotional amplification of cancer-related uncertainty and distress. Because these processes were not directly measured in the present study, they should be considered plausible explanatory mechanisms rather than demonstrated mediators.

The improvement in sleep problems may involve both direct and indirect pathways. Sleep disturbance in patients with advanced cancer is closely related to psychological symptoms and often requires multifaceted management (34–36). Reductions in anxiety and depressive symptoms may lessen pre-sleep worry and cognitive arousal, while body-scan and breathing-based practices may promote relaxation and reduce physiological arousal. Previous evidence that mindfulness-based programs can improve sleep quality in cancer populations further supports a potential link between mindfulness practice and sleep-related outcomes (37). These complementary pathways may help explain why improvements in sleep problems accompanied improvements in psychological symptoms. By contrast, the findings for pain intensity were more limited. Although NRS scores decreased over time in both groups, the between-group difference in change was not statistically significant. This pattern is consistent with a recent meta-analysis in palliative care, in which mindfulness-based interventions showed clearer pooled benefits for anxiety and depression than for pain (38). These findings suggest that pain-related effects may be less consistent than psychological benefits and may depend on intervention characteristics and clinical context. Cancer-related pain is multifactorial and generally requires multimodal management incorporating appropriate pharmacological treatment together with psychological, non-pharmacological, and self-management approaches (39–42). Therefore, micro-mindfulness should be viewed as a supportive strategy for emotional regulation and symptom coping rather than a substitute for standard pain management.

The responder analysis further suggests that patient characteristics may influence the benefit obtained from micro-mindfulness. Patients with higher monthly household income were more likely to achieve psychological symptom response, which may reflect differences in access to supportive resources, stability of the care environment, and capacity for self-management (43, 44). In contrast, patients with ECOG performance status ≥2 had lower odds of response, suggesting that poorer physical function and greater disease burden may limit engagement with or benefit from self-directed psychological practices (45). Digestive system cancers were associated with lower odds of response in univariable analysis but not after adjustment, suggesting that this association may be partly explained by overall disease burden or functional status. These exploratory findings suggest that patients with poorer physical condition or limited socioeconomic resources may require more intensive or individualized psychological support. Caregiver-supported approaches may also warrant further evaluation in palliative care, given emerging evidence that mindfulness-based interventions involving informal caregivers can reduce caregiver burden and burnout (46).

This study has several clinical implications. The embedded micro-mindfulness program was designed to be brief and repeatable, with a format intended to facilitate integration into routine palliative care. It can be introduced at the bedside by trained nurses or physicians, supported by audio materials, and continued after discharge through online follow-up. In the present study, all participants in the MM group completed the 28 scheduled practice sessions over 4 weeks, indicating high documented adherence to the program. Consistent with implementation-focused research emphasizing the importance of context-specific adaptation, accessible patient materials, and healthcare-professional support when integrating brief mindful-breathing practices into palliative care, the present program incorporated standardized bedside guidance, audio materials, and post-discharge follow-up as part of its delivery strategy (47). This delivery model is also consistent with broader efforts to integrate supportive and palliative approaches into routine nursing and clinical care (48). However, adherence alone does not establish overall feasibility or acceptability, and participant satisfaction and perceived burden were not formally assessed in the present study.

Several limitations should be acknowledged. First, this was a single-center study, and the findings may not be fully generalizable to other institutions or care settings. Second, the follow-up period was relatively short, and the long-term sustainability of symptom improvement remains unclear. Third, most outcomes were based on patient-reported scales, which may be influenced by subjective perception and response bias. Fourth, although baseline characteristics were generally comparable between groups, the prospective non-randomized observational design and voluntary participation in the micro-mindfulness program may have introduced self-selection bias and residual confounding. Patients who chose to participate may have differed from non-participants in unmeasured characteristics, which could have contributed to the observed between-group differences. Fifth, the study was not prospectively registered, which may limit transparency regarding the prespecified outcomes and analyses. Finally, although practice adherence was documented, acceptability, participant satisfaction, perceived burden, caregiver-related outcomes, and provider-level implementation outcomes were not systematically assessed. Future randomized multicenter studies with longer follow-up are needed to strengthen causal inference and determine the durability of the observed improvements. Further research should also determine the optimal duration, frequency, and component structure of brief mindfulness practices and identify which patients are most likely to benefit.

## 5 Conclusion

Embedded micro-mindfulness was associated with greater improvements in anxiety and depressive symptoms, with additional benefits for psychological distress and sleep problems, among patients with advanced cancer receiving palliative care. These findings support further evaluation of this brief program as a supportive-care strategy in palliative care.

## Data Availability

The raw data supporting the conclusions of this article will be made available by the authors, without undue reservation.
